# Effects of change in dysfunctional beliefs and self-esteem in avatar-based cognitive therapy for symptoms of social anxiety disorder: a randomized parallel trial

**DOI:** 10.1038/s41598-026-39641-x

**Published:** 2026-02-12

**Authors:** Nicolina Laura Peperkorn, Julia Ohse, Janosch Fox, Sarah Limberg, Bakir Hadžić, Matthias Rätsch, Jan-Niklas Voigt-Antons, Michael Witthöft, Youssef Shiban

**Affiliations:** 1 Clinical Psychology Department, PFH Private University of Applied Sciences, Weender Landstraße 3-7, 37073 Göttingen, Germany; 2 ViSiR, Reutlingen University, Alteburgstraße 150, 72762 Reutlingen, Germany; 3 HSHL Hochschule Hamm-Lippstadt, Marker Allee 76-78, 59063 Hamm-Lippstadt, Germany; 4 Faculty of Psychology, Ruhr University Bochum, Massenbergstraße 9-13, 44787 Bochum, Germany

**Keywords:** Avatar therapy, Digital intervention, Cognitive therapy, Dysfunctional beliefs, Social anxiety disorder, Subclinical social anxiety disorder, Social phobia symptoms, Diseases, Health care, Psychology, Psychology

## Abstract

Social anxiety is both highly prevalent and debilitating, with dysfunctional beliefs playing a crucial role in its persistence. This randomized controlled trial examined whether a remote avatar-based intervention for restructuring dysfunctional beliefs was associated with changes in social anxiety symptoms in a sample with subclinical social anxiety disorder (SAD). Data from *N =* 235 participants who completed all study stages were included in the analyses. While participants in the intervention group were tasked with contradicting their personal dysfunctional beliefs verbalized by the avatar, participants in the control group contradicted nonsense statements uttered by the avatar. Three sessions took place over three consecutive days. Data collection occurred pre-, post-intervention, and at a 14-days-follow-up. A significant group x time interaction effect (*F*(1, 233) = 4.106, *p* < .05) indicates a stronger SAD symptom reduction within the intervention group in comparison to the control group. Post-hoc analysis revealed a medium effect size for symptom reduction in the intervention group (*d* = 0.57) and a small effect size for the control group (*d* = 0.34). An additional linear multiple regression analysis identified that increased self-esteem (*B* = − 0.56, *p* < .001) was associated with reductions in SAD symptoms. An increase in dysfunctional social phobia cognitions was associated with an increase in SAD symptoms (*B* = 0.175, *p* = .021). Avatar-based cognitive restructuring was associated with reductions in SAD symptoms in a subclinical sample. Changes in self-esteem and social phobia-related cognitions were also associated with symptom changes, but causal relationships cannot be inferred from this study design.

**Supplementary Information:**

The online version contains supplementary material available at 10.1038/s41598-026-39641-x.

## Introduction

Social anxiety disorder (SAD) is one of the most common mental disorders, with a lifetime prevalence of about 7%^1^. Individuals with SAD experience considerable impairments in their ability to form and maintain social and romantic relationships, as well as in their academic and occupational performance^1^. In addition, SAD is linked to early school dropouts and a heightened risk of unemployment^2,3^. Even subclinical levels of social anxiety, which occur in 20% of the general population, pose a substantial individual burden^4^. As remission rates for SAD vary strongly^5,^ a closer inspection of theoretical models explaining the maintenance of this disorder, as well as therapy methods tackling model components, is well warranted.

The cognitive models of SAD, such as those proposed by Clark and Wells^6^, provide a valuable theoretical framework for understanding its maintenance processes. A shared constituent of cognitive models is that SAD is maintained by dysfunctional information processing. Dysfunctional beliefs about oneself and the social environment are factors within this dysfunctional information processing, as they lead individuals with SAD to interpret social situations as threatening^7^. In particular, automatic negative thoughts concerning perceived judgments by others are believed to contribute to the maintenance of SAD, often leading to the individual’s attention focusing on the self and/or cognitive representations of the self. Within the cognitive model of SAD^6^, these automatic thoughts increase physical anxiety symptoms and reinforce safety behaviors, which in turn aggravate negative evaluations of the self in social situations and also lead to an avoidance of such situations, meaning the negative evaluations of the self cannot be disproved with facts. Due to their influence on multiple factors within the model, automatic thoughts appear to be a promising target for therapy. Another shared component of cognitive models of social anxiety is the negative evaluation of the self/ perception of the self as defective^6,8,9^. Stopa^7^ highlighted this similarity in particular and pointed out the importance of self-structure, while Moscovitch^10^ outlined the exposure of self-attributes perceived as flawed as the core component of social anxiety disorder. Hulme et al.^11^ proposed that the complex experiences of the self impacted by SAD are measurable in the construct “self-esteem”. According to their logic, the concept of self-esteem captures numerous aspects of the working self while still being easily measurable. The connection between SAD and low self-esteem was empirically validated by Iancu et al.^12^, who found individuals with SAD to generally exhibit low self-esteem, as well as by Harris and Orth^13^ who found self-esteem to be a significant predictor of social anxiety. Additionally, multiple other studies found a close connection of SAD and low self-esteem (e.g^14–16^. Self-esteem in general is an important transdiagnostic factor and was therefore evaluated as a secondary outcome by Kindred et al.^17^ in their meta-analysis of long-term cognitive-behavioral therapy (CBT) outcomes for social anxiety disorder.

Within such CBT frameworks, automatic thoughts and dysfunctional cognitions (negative evaluations of the self) are tackled through cognitive restructuring. Cognitive restructuring describes a process, in which maladaptive automatic thoughts leading to anxiety, avoidance, and negative self-perception, are identified, questioned and subsequently replaced with more functional alternatives. Not just Kindred et al.^17^ but further meta-analyses reveal CBT to be an effective treatment^18^ reducing SAD symptoms and improving overall functioning and quality of life. Evidence points towards CBT’s effectiveness extending well beyond the face-to-face context: A meta-analysis by Winter et al.^19^ revealed large effect sizes for remote CBT methods for treating SAD. Most of these treatments use a form of cognitive restructuring, delivered via internet, self-help guides (bibliotherapy), application, or videoconference.

A study by Kimani et al.^20^ investigated the potential of a virtual agent for cognitive restructuring of automatic thoughts arising in people with public speaking anxiety. Public speaking anxiety is a subtype of social anxiety disorder^21^. The virtual agent in Kimani et al.’s study^20^ coached *n* = 13 participants in the treatment group before they held a presentation, by offering functional alternatives to maladaptive thoughts on public speaking. In comparison to the control group (*n* = 11), a stronger reduction in dysfunctional thoughts was reported for the treatment group. Given the large number of variables tested in a relatively small sample, this warrants further investigation. Our approach aims to extend the investigation into cognitive restructuring for social anxiety, delivered by a virtual agent. Contrary to Kimani et al.^20^, where the virtual agent appears as a coach for the participants, the virtual agent within our study poses as an embodiment of the maladaptive automatic thoughts. Hence, participants are tasked with contradicting the agent uttering their maladaptive thoughts with functional, alternative statements. This approach has previously been tested for dysfunctional cognitions in in-patients currently receiving depression therapy in a clinic^22^, in participants with subclinical depressive symptoms^23^ and in participants with subclinical eating disorder symptoms^24^. Results from these studies suggest that avatar-based interventions may hold promise for subclinical mental illness, even when focused on a different mental disorder.

Based on said promising results, we hypothesize the following:

1. The intervention group will experience a significantly greater decrease in SAD symptoms throughout the intervention compared to the control group.
2. Based on the cognitive model of SAD we hypothesize:
  - A reduction in automatic dysfunctional thoughts leads to a decrease in SAD symptoms.
  - An increase in self-esteem is associated with a decrease in SAD symptoms.

## Methods

### Study design and outcomes

The study was conducted as an experimental design with two parallel treatment groups (intervention vs. control). Participants were randomly allocated to either of these treatment groups in a 1:1 ratio. Measurement took place at three time points: Prior to the intervention (pre-treatment), after the last treatment session (post-treatment), and 14 days after the intervention (follow-up). Follow-up was defined a priori as the primary endpoint, as it may capture enduring changes in symptom severity, whereas post-intervention assessments were designated as secondary/exploratory endpoints, reflecting short-term effects that may be transient. The following questionnaires were used for the analyzed outcome variables ‘social anxiety symptom severity’, as well as ‘self-esteem’, and the intensity of ‘social phobia cognitions’: the German version of the Social Phobia Inventory (SPIN)^25^, Rosenberg Self Esteem Scale (RSES)^26^, and Social Cognitions Questionnaire (SPK)^27^.

#### Materials

To assess whether participants met the inclusion criterion of subclinical SAD symptom strength, the German version of the mini-SPIN (Mini - Social Phobia Inventory) was employed. With its three items rated on a 5-point Likert-scale, the mini-SPIN is the short version of the SPIN (Social Phobia Inventory) and was chosen for its economic properties^28^. Psychometric properties show a good internal consistency (*α =* 0.80 − 0.83) and test-retest reliability is *R =* .61. A cut-off score of 4 was found to yield the best balance of sensitivity and specificity within the German population^29^. Therefore, a mini-SPIN-score of 4 and above was chosen as an inclusion criterion for this study.

Furthermore, the German version of the SPIN was employed as a self-assessment questionnaire of symptom severity. While the short version of this measure was used as a screening tool, prior to the actual investigation, the full 17-item version of the SPIN was used to determine SAD symptom severity and validate the inclusion of symptomatically burdened participants. The SPIN consists of the subscales ‘anxiety in different situations’, ‘avoidance in different situations’, and ‘physiological symptoms’, and can be processed in ten minutes^30^. Agreement with the items is scored on a five-point Likert scale (0 = Not at all; 1 = A little; 2 = Quite a bit; 3 = Very strongly; 4 = Extremely), leading to a total score ranging from 0 to 68^25^. The observation period of the SPIN refers to the past seven days^31^. Psychometric properties include reliability parameters ranging from good to very good, with an internal consistency of *α =* .95 and a retest reliability of *R =* .91^30^. The tool was found to efficiently differentiate between people with and without SAD, as well as between different levels of SAD severity^25^. A cut-off score of 19 has been shown to distinguish individuals with social phobia from non-anxious individuals^44^. Accordingly, a SPIN-score of 19 or above was used as an inclusion criterion in our study. Additionally, the measure is sensitive to potential declines in symptoms over time and can therefore be utilized to monitor response to treatment^25^.

The RSES from von Collani and Herzberg^32^, based on the original version from Rosenberg^26^, was used as a self-report measure to assess subjects’ self-esteem. The test includes ten statements to assess the expression of positive or negative self-esteem on a 4-point Likert scale (from ‘do not agree at all’ = 1 to ‘fully agree’ = 4), leading to a sum score between 0 and 30. A sum score below 15 is an indicator for low self-esteem. The scale shows a satisfying internal consistency with Cronbach’s Alpha = 0.85^32^.

In addition, the German version of the Social Cognitions Questionnaire^33^, the “Fragebogen zu sozialphobischen Kognitionen” (SPK)^27^, was employed. This self-report measure contains 22 items, with each item representative of a belief that is typically related to social anxiety (e.g. ‘People will not like me’). These 22 beliefs are rated on two scales with the first scale assessing the average frequency of the thought on a 5-point Likert scale ranging from 1 (‘The thought never occurs’) to 5 (‘The thought always occurs when I am nervous’), and the second scale capturing the average conviction rating for the belief from 0 (‘I do not believe in this thought’) to 100 (‘I am totally convinced that this thought is true’^27^. These items can be categorized into three scales: ‘Negative self’, ‘Fear of failure’, and ‘Fear of showing visible body symptoms’^33^. The reliability parameters of the individual scales and the overall scale are in the good to very good range. The internal consistency and test-retest-reliability of the SCQ for adults are described as good by the authors^34,35^. The test was used in measuring symptom differences in treatment studies and had the ability to depict changes in social anxiety belief ratings which indicates that the test is sensitive to change^36–38^.

### Intervention

The intervention consisted of three components: (a) psychoeducation, (b) formulation of dysfunctional cognitions and alternative assumptions, and (c) the avatar session. All participants received psychoeducation about dysfunctional cognitions, which was delivered digitally to them as an illustrated text document for self-study. The psychoeducational text concluded with the task to formulate three individual dysfunctional cognitions and three corresponding alternative functional assumptions. Following this procedure, participants could start the avatar session by accessing the automated web application in a browser of their choice. The structure of the intervention closely followed the procedure established in Fey et al.^24^ and Peperkorn et al.^23^: Participants in the experimental group were subsequently confronted with each of their three individual dysfunctional beliefs in three rounds over the course of one session. Their task was to verbally contradict each of these dysfunctional beliefs, right after it was uttered. Each session lasted between 10 and 15 min. In contrast, the participants in the control group were confronted with false statements (e.g., ‘Hamburg is the capital of Germany’), and tasked with contradicting them (e.g., ‘No, this is wrong. Berlin is the capital of Germany’). In contrast to the approach of Kocur et al.^22^, participants had the flexibility to initiate the intervention themselves by accessing the application via their web browser. Therefore, the intervention session took place without the presence of an experimenter.

**Fig. 1:**
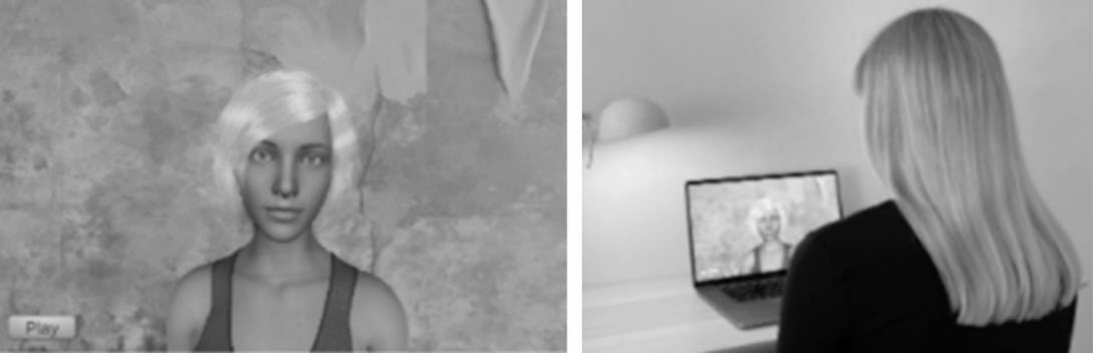
Experimental setup. *Note.* The image on the left displays the virtual avatar as presented at the start of the intervention. Participants can initiate the confrontation phase by pressing the “Play” button on the interface. The image on the right illustrates the experimental setup, where the participant is seated in front of a computer screen displaying the interface with the virtual avatar.

The digital intervention was created in the WebGL version of the VR development platform Unity, with the Unity3D engine generating the application itself. The avatar was realized with the Genesis 8 model from Daz 3D, speech animations were realized with the SALSA LipSync v2 Suite. The avatar was rendered from head to torso and was presented as a female Caucasian, around 20–40 years old and dressed in casual clothes. Dysfunctional beliefs and false statements were expressed in a neutral tone of voice and with a neutral facial expression while maintaining eye contact with the participants. The experimental setup can be seen in Fig. 1.

### Ethics

This study was preregistered on the German Clinical Trials Register (DRKS) on 06/09/2024, under the DRKS-ID: DRKS00035005. Prior to their participation, respondents were provided with written details outlining the study’s purpose, the intervention and questionnaire procedures. Additionally, participants received information on data collection, storage, and protection. Before entering the experiment, participants’ consent for participation, data collection, and processing was obtained in accordance with the Declaration of Helsinki. The Ethics Committee of the PFH Private University of Applied Sciences Göttingen gave their approval for the study protocol (reference number 251981/3) before data collection began. The study complies with GDPR regulations, ensuring that all data are managed in a transparent, ethical, and secure manner. Participants were also informed of their rights to access, amend, delete, or restrict the use of their personal data.

### Procedure

The study was multi-phased, including a two-stage screening process, a pre-intervention assessment, three sessions of avatar-based interventions, a post-assessment and a follow-up-assessment which took place 14 days after the last session. All questionnaires and interventions were provided online, granting participants the flexibility to choose their preferred time and place to complete them within the study schedule. Participants were recruited via email lists, social media platforms, and university forums, with informed consent obtained from each individual. The study began with a pilot phase on August 1, 2024, during which study procedures were tested and data collection processes were piloted. Only participants recruited after the formal registration of the study in the German Clinical Trials Register on September 6, 2024, were included in the primary analysis.

After recruitment, a two-step screening was carried out to identify a subclinical sample with social anxiety. The first step of the screening included a short socio-demographic questionnaire, as well as the mini-SPIN^28^, to verify participants met the inclusion criteria and did not meet any exclusion criteria. Additionally, demographic data on age, gender and highest level of education were gathered. At this stage, two variables were defined as inclusion criteria: A minimum age of 18 years and social anxiety score of 4 and above on the mini-SPIN. Participants meeting these first screening criteria moved forwards to the second screening step, in which the complete 17-item version of the SPIN was employed, with a score greater than 19 as inclusion criterion to ensure the presence of subclinical social anxiety^44^. Exclusion criteria were visual/ auditory impairments that would negatively impact participation, insufficient proficiency of the German language, former psychotic or manic episodes as well as current involvement in psychotherapeutic treatment, to ensure (a) the subclinical nature of the sample, (b) the potential to fully understand questionnaires and the intervention, and (c) that potential changes in the outcome variables were not due to such psychotherapeutic influences.

Eligible participants were randomly assigned to either the intervention or the control group via an online tool, with no stratification by gender or other demographic variables. After this allocation, participants received a standardized email containing a link to either the experimental intervention or the control condition. Participants did not receive information on the existence of two distinct intervention groups and/or their specific group assignment. While the experimenters sending out the emails were aware of the group assignments, they did not have direct contact with the participants that went beyond sending out standardized text mails. Since the avatar-intervention took place in an automated web application, no experimenter was present during this process.

During pre-measurement, participants filled in the self-assessment questionnaires SPIN, RSES and SPK. Additionally, a digital psychoeducation session on dysfunctional beliefs using explanatory images and texts was provided to both groups. The session is grounded in the theoretical framework of Beck’s cognitive model^39^ and its adaptations for social anxiety (e.g,^40,41^. , providing participants with a theoretically informed understanding of dysfunctional cognitions, including how they arise and influence perception. It was embedded within the survey and guided by an illustrative figure, which presented the concepts with practical examples. The psychoeducational session was designed to raise participants’ awareness for their individual dysfunctional beliefs. Next, participants were instructed to write down three of their individual dysfunctional beliefs. Following the completion of the self-assessment questionnaires, the first avatar intervention session took place. In this session, participants were confronted with each of their three dysfunctional beliefs three times and tasked with contradicting them. On the consecutive two days, the second and third intervention sessions took place, following the 3 × 3 scheme established in session one. Post-measurement took place directly after the last avatar session, once more featuring the self-assessment tools SPIN, RSES and SPK. Fourteen days after the last session, the follow-up-measurement took place, once again measuring outcome variables with the SPIN, RSES and SPK. A detailed overview of this study procedure is presented in supplementary Fig. 1.

### Statistical analysis

The statistical analysis was conducted using R 4.0.4. The directional hypothesis H1 was tested using a two-factor repeated measures analysis of variance (2 × 2 ANOVA), with the between-subjects factor *treatment group* (intervention group [*n* = 118] vs. control group [*n* = 117]) and the within-subjects factor *time* (pre-intervention and follow-up). The dependent variable for the hypothesis was social phobia symptom strength, as measured by the SPIN-score. Follow-up was treated as the primary outcome time point in confirmatory analyses. Post-treatment assessments were considered secondary/exploratory and were therefore not included in the ANOVA. The decision to not include post-treatment as a within-subjects factor was made a priori during preregistration. This decision was based on the rationale that changes observed immediately after the final session may reflect short-term or transient effects, rather than meaningful or enduring symptom change.

Additionally, a linear regression analysis was performed to examine the predictors of change in social phobia strength (SPIN) from pre-intervention to follow-up. Based on the proposal of Hulme et al.^11^ to measure multiple aspects of the negative working self by evaluating self-esteem, this model included self-esteem change (RSES) and change in social phobia cognitions (SPK) as independent variables.

We decided to not perform an intention-to-treat analysis (ITT), since the dropout rate was too high to reliably restore missing data with imputation methods. While a systematic outlier detection was performed based on the IQR method, it was decided not to exclude any participants as high scores on the measurements employed reflect a higher symptomatic burden.

## Results

### Sample

The required sample size for this study was determined a priori using the program G*Power^42^. Since effect sizes in subclinical populations are generally smaller than in clinical samples^43^, and with reference to previous studies by Kocur et al.^22^, Fey et al.^24^ and Peperkorn et al.^23^, we estimated an effect size of Cohen’s *f =* 0.15. With a required power (1 - β) of 0.95 and an α-error probability of 0.05, the power analysis indicated that a total of *N* = 148 participants would be necessary to test our hypotheses.

A sample of *n* = 2115 respondents was screened, of whom, *n* = 1350 participants were excluded. Among these, *n* = 531 (25.1% of the total sample) participants met one or more predefined exclusion criteria, including current involvement in psychotherapeutic treatment (*n* = 148), a history of psychotic or manic episodes (*n* = 348), visual or auditory impairments (*n* = 20), or insufficient proficiency in German (*n* = 15). The remaining *n* = 819 participants were excluded due to incomplete or invalid responses, such as not completing the screening or only opening the survey link.

In addition, *n* = 254 participants were excluded, due to not meeting the inclusion criterion of a Mini-SPIN-score ≥ 4 and a SPIN-score of ≥ 19. This resulted in *n* = 511 participants being enrolled and randomized to either the intervention or control group. Of these, *n* = 276 participants did not complete all stages of the experiment from pre-measurement to final data collection, corresponding to a dropout rate of 54%. This resulted in a total sample of *N* = 235 participants, which was analyzed for this study. A detailed overview of participant progress, including the number of exclusions and dropouts, is provided in supplementary Figure S2. Despite an allocation ratio of 1:1 for intervention and control group and drop-out, numbers within each group were comparable: Intervention group (*n* = 118, f: 81, m: 33, d: 1, *M*_age_ = 32.9, *SD*_age_ = 13.5) or the control group (*n* = 117, f: 80, m: 31, *M*_age_ = 32.2, *SD*_age_ = 12.7). Regarding the highest educational qualification, having a completed university degree was dominant with *n* = 94 subjects. Sociodemographic characteristics can be found in Table 1. Due to technical issues, part of the sociodemographic data (gender, age, education) was missing for *n* = 9 participants. Pre-intervention scores for SPIN, RSES, and SPK, including all randomized participants (*N* = 511), are presented in Table 3.

**Table 1:** Sociodemographic characteristics of the sample.

	EG		CG		Total sample	
	*n*	%	*n*	%	*n*	%
Gender						
Female	81	70.43	80	72.07	161	71.2
Male	33	28.70	31	27.93	64	28.3
Diverse	1	0.87	0	0	1	0.44
Education						
University degree	44	38.26	50	45.05	94	41.84
University qualification	37	32.17	30	27,03	67	29.65
Completed training	24	20.87	26	32.43	50	22.12
Other	10	8.70	5	4.50	15	6.64

Note. EG = intervention group. CG = control group. *N* = 235.

**Table 2:** Descriptive metrics for SPIN-, RSES-, and SPK-scores.

Variables	EG		CG		Baseline Comparison (*p*-Value)
	M	SD	M	SD	
SPIN					
Pre-Intervention	36.0	12.0	32.1	9.64	0.008
Post-Intervention	27.0	16.0	26.5	14.2	
Follow Up	28.3	13.1	27.5	13.6	
RSES					
Pre-Intervention	15.7	5.95	15.4	6.10	0.693
Post-Intervention	14.7	7.86	14.9	7.31	
Follow Up	17.7	6.02	16.5	6.95	
SPK					
Pre-Intervention	57.7	19.3	51.9	16.9	0.014
Post-Intervention	45.9	24.6	47.4	21.8	
Follow Up	48.4	17.5	49.6	19.5	

Baseline (pre-intervention) differences between the intervention group (EG) and control group (CG) were assessed using independent-samples Welch t-tests, with p-values reported only for baseline comparisons. Post-intervention and follow-up values are descriptive, and are not analyzed for between-group differences in this table.

Note: EG = intervention group. CG = control group. *N* = 235.

**Table 3:** Pre-intervention SPIN-, RSES-, and SPK-scores for all randomized participants (*N* = 511), including dropout (*N* = 276).

Variables	EG		CG	
	M	SD	M	SD
SPIN				
Pre-Intervention	35.8	11.5	32.9	10.6
RSES				
Pre-Intervention	15.8	4.32	15.7	4.26
SPK				
Pre-Intervention	57.1	19.6	53.7	19.0

Note: EG = intervention group. CG = control group.

### Changes in social anxiety symptoms

#### Descriptive data

**Fig. 2:**
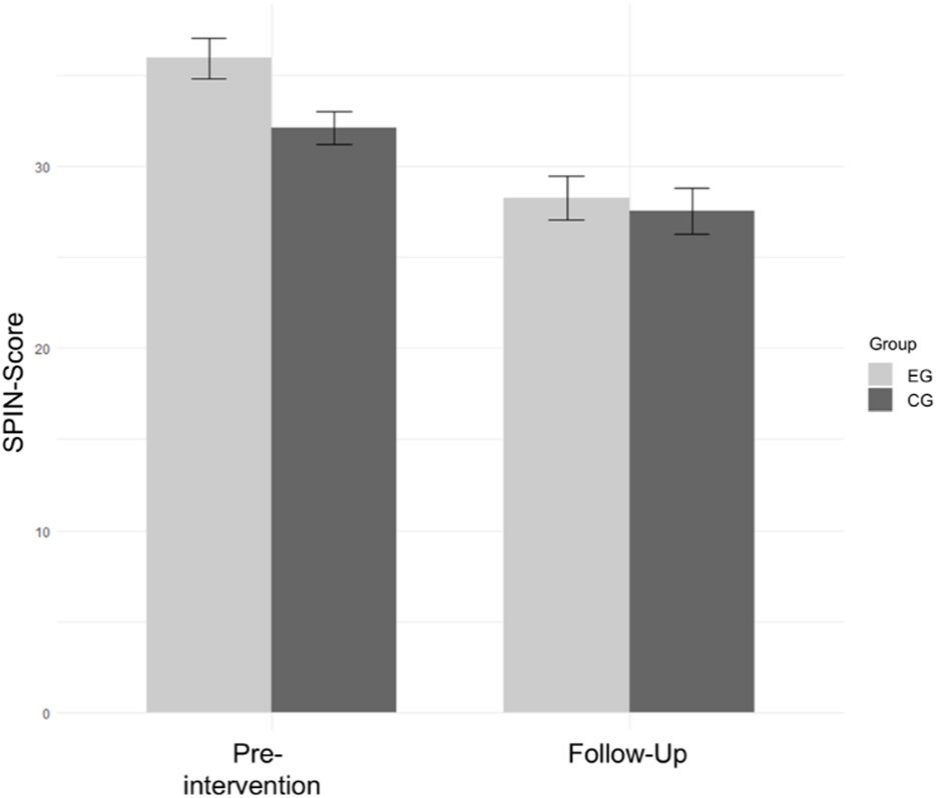
SPIN-scores for the intervention and control group pre-intervention and follow-up. *Note*. Mean scores of the Social Phobia Inventory (SPIN) for the intervention group (EG; *n* = 118) and the control group (CG; *n* = 117). Error bars show +/-1 standard errors.

As shown in Fig. 2 the pre-intervention SPIN-score was *M* = 36.0 (*SD* = 12) for the intervention group (*n* = 118) and *M* = 32.1 (*SD* = 9.64) for the control group (*n* = 117). According to the cut-off values proposed by Ranta et al.^44,^ a score of 19 or higher indicates the presence of symptoms to the extent of SAD. The statistical analysis revealed a significant difference in pre-intervention SPIN-scores between the groups with higher scores in the intervention group (Welch two-sample t-test: *t*(223.26) = 2.70, *p* = .008). At follow-up, the mean SPIN-score in the intervention group was *M* = 28.3 (*SD* = 13.1), while the mean score in the control group was *M* = 27.5 (*SD* = 13.6), as depicted in Table 2, showing a decrease of 7.7 points within the intervention group and a decrease of 4.6 points within the control group.

Post-hoc analysis revealed a medium effect size for decrease in SAD symptoms within the intervention group (*d* = 0.57) and a small effect size for decrease of SAD symptoms in the control group (*d* = 0.34).

#### Inference statistics

A 2 × 2 repeated measures ANOVA, analyzing *treatment group* as a between-subjects factor, and *time* as a within-subjects factor, found a statistically significant interaction effect for SAD symptom severity (SPIN, *F*(1, 233) = 4.106, *p* < .05, *η2* = 0.004). A significant main effect for the within-subjects factor *time* was revealed, *F*(1, 233) = 65.597, *p* < .001, *η2* = 0.060. However, no effect was found for the factor *group*, *F*(1, 233) = 2.700, *p* = .102, *η2* = 0.009. A visualization of these results can be seen in Fig. 2.

#### Exploratory analysis

As a manipulation-check a 2 × 2 repeated measures ANOVA on SPK-scores was conducted, with *treatment grou*p as a between-subjects factor and *time* as a within-subjects factor. There was no significant main effect for the factor *group*, *F*(1, 233) = 0.83, *p* = .363, *ges* = 0.003, but a significant main effect for the factor *time*, *F*(1, 233) = 36.38, *p* < .001, *ges* = 0.037, and a significant Group × Time interaction, *F*(1, 233) = 7.44, *p* = .007, *ges* = 0.008. This indicates that the reduction of dysfunctional thoughts differed between the groups over time. Paired t-tests revealed that the experimental group (EG) exhibited a moderate reduction in SPK-scores (Cohen’s *d* = 0.60, *t*(117) = 6.53, *p* < .001), whereas the control group (CG) showed a smaller reduction (Cohen’s *d* = 0.21, *t*(116) = 2.23, *p* = .028).

### Relation of self-esteem and cognitive changes to social phobia symptoms

#### Inference statistics

A linear regression analysis was conducted to examine the predictors of change in SAD symptom severity (SPIN) from pre-intervention to follow-up. The model included self-esteem change (RSES) and change in social phobia cognitions (SPK) as independent variables.

The overall model was statistically significant, *F*(2,232) = 6.616, *p* = .001, explaining approximately 5.40% of the variance in SPIN change, as indicated by the multiple R-squared value (*R*^2^ = 0.054).

The results revealed several significant predictors. Specifically, self-esteem change was negatively associated with change in SAD symptom severity (*B* = -0.448, *β* = -0.265, *p* < .001). Additionally, change in social phobia cognitions (SPK) was positively associated with change in SAD symptom severity (*B* = 0.103, *β* = 0.187, *p* = .013), indicating that increases in the frequency of social phobia cognitions corresponded with higher symptoms in SAD at follow-up. In contrast, the residuals of the model indicated a minimum of -57.92 and a maximum of 37.26, with a median of 0.776. The residual standard error was 11.39 (Table 4).

**Table 4:** Results of linear regression.

Effect	B	SE	t	*p*	95% CI		β
					LL	UL	
Intercept	-5.602	0.802	-6.985	< 0.01	-7.182	-4.022	
Change in RSES	-0.448	0.126	-3.552	< 0.01	-0.696	-0.199	-0.265
Change in SPK	0.104	0.041	2.504	0.01	0.022	0.184	0.187

*Note. N* = 235, *R*^*2*^ = 0.05, adjusted *R*^*2*^ = 0.05, *F*(11.39, 232) = 6.62, *p* < .01, Standardized coefficients are not reported for the intercept.

## Discussion

The primary aim of this study was to investigate whether a transdiagnostic tool for the modification of dysfunctional cognitions using CBT-elements (cognitive restructuring) is associated with changes in social phobia symptoms in individuals with subclinical social anxiety. The results supported the initial hypothesis. In addition, our findings indicated that increases in self-esteem were associated with reductions in social anxiety symptoms, whereas increases in dysfunctional social phobia cognitions (SPK) were associated with symptom increases (*B* = 0.175, *p* = .021). The regression model including changes in self-esteem and SPK as predictors was statistically significant (*F*(2,232) = 6.616, *p* = .001), explaining approximately 5.4% of the variance in SPIN change. Although this R^2^ value is relatively small, which is common in studies involving subclinical samples, these findings are consistent with the theoretical framework that changes in self-esteem and dysfunctional cognitions may be associated with symptom improvement. However, when interpreting these results, it should be considered that other factors may also contribute to the observed effects and self-esteem was not directly targeted in the intervention. Therefore, based on the study design, causal interpretations cannot be made.

This study aimed to extend the findings of Kimani et al.^20^, who used a virtual coach to help reduce speech anxiety through psychoeducation and cognitive restructuring. We combined this approach with the methodology from Kocur et al.^22^, where participants confronted their dysfunctional beliefs using a virtual avatar to assess the effectiveness of the Avatar intervention for individuals with social anxiety. Kimani et al.^20,^ further showed that a virtual coach combined with cognitive restructuring could reduce dysfunctional thoughts associated with speech anxiety. Our findings are consistent with these studies, as the experimental group in this study experienced a greater reduction in social phobia symptoms compared to the control group, suggesting that repeated engagement with avatar-based exercises may be associated with reductions in social phobia symptoms.

The positive relationship between self-esteem increase and social phobia symptom decrease is in line with the suggestion of Bögels and Mansell^45^, that self-esteem is one of six overlapping factors for change in the treatment of social anxiety. However, the direction of this relationship remains uncertain, as changes in self-esteem may occur concurrently with the improvement in SAD symptoms rather than representing a causal mechanism.

While the study provides valuable insights, several limitations should be acknowledged. First, the sample was not representative of individuals with lower educational backgrounds, which limits the generalizability of the results to a broader population. Second, there was a gender imbalance, with female participants being overrepresented. Although women are more frequently affected by social phobia, this overrepresentation may have influenced the outcomes. Future research should strive for a more balanced sample to better reflect the diversity of those affected by social anxiety. Addressing these limitations will help strengthen the validity and applicability of findings in future studies.

Additionally, the observed gender distribution reflects the individuals who met the inclusion criteria and completed the study, as participants were randomly assigned to the intervention and control groups without stratification or targeted recruitment by gender. Therefore, gender was collected as a descriptive sociodemographic variable and was not included in the analyses. Future research could take this into account by including gender as an additional factor in the analysis, which could test potential gender-specific effects and improve generalizability.

Furthermore, the pre-values of the SPIN and SPK differed significantly between the experimental and control group. The significant differences in the pre-scores indicate that the two groups were not comparable before the start of the intervention. This difference could contribute to regression-to-the-mean effects, limiting the interpretation of the intervention’s effects. Observed changes may therefore reflect not only the effect of the intervention but also initial group differences. As the experimental group already had higher SPIN-scores before the intervention, this could influence the interpretation of the effectiveness of the intervention. For example, a greater reduction in symptoms in the experimental group could be partly due to higher baseline scores, leaving more room for improvement. At the same time, these higher baseline scores suggest that participants in the experimental group were more severely affected and potentially more difficult to treat. Despite this, the ANOVA revealed a statistically significant intervention effect, though the very small effect size suggests caution in interpretation. Taken together, higher baseline severity may have allowed more room for improvement but could also have limited the ease of achieving change, highlighting both a potential advantage and a challenge when interpreting intervention outcomes.

While dropout rates were comparable between groups, attrition remains a challenge in fully remote interventions and may further limit interpretation of outcomes, particularly for participants with higher baseline symptom severity. Therefore, results should be interpreted considering this limitation. For future studies, revising the randomization procedure to minimize baseline differences and ensure more comparable groups may help reduce potential bias.

Another limitation of our design is that we did not include post-intervention outcomes in the confirmatory analyses. This decision was made a priori at the time of preregistration and therefore did not constitute a deviation from the study protocol. The rationale for this approach was that immediate post-intervention assessments might be confounded by confrontation-related distress and short-term experimental influences. By focusing on the change from pre–intervention to follow-up, we prioritized a conservative estimate of sustained therapeutic effects. However, this approach restricts conclusions about the immediate mechanisms of change, which remain an open question for future studies.

In addition, the observed interaction effect has a very small effect size (*η*^2^ = 0.004). Therefore, the results should be interpreted with caution and regarded as preliminary evidence rather than strong causal proof.

Another limitation is the absence of a true control group that received no intervention. Although the control group engaged with general false statements, they also participated in disputing exercises and psychoeducation, which may explain their symptom improvement. Time and increased awareness of cognitive distortions could have contributed to these changes, even without direct confrontation with dysfunctional beliefs^46^.

Finally, although changes in self-esteem were associated with reductions in social anxiety symptoms, the study design does not allow causal conclusions. Self-esteem was not directly targeted. Future research could address this by experimentally manipulating self-esteem or examining the timing of symptom and self-esteem changes to clarify its potential role as a mechanism of intervention effects.

It should be noted that the observed improvements may not be solely attributable to psychoeducation and cognitive restructuring. Prior studies have shown that avatar-based or virtual exposure can elicit anxiety and function as exposure therapy for social anxiety disorder. For instance, Powers et al. demonstrated that VR conversations with a digital avatar elevated fear rating, underscoring the exposure potential of such methods^47^. In our design, both the intervention and control group interacted with the avatar, ensuring that all participants experienced a comparable level of exposure to the virtual agent. This design allowed us to control for the general exposure effects and specifically examine the added contribution of engaging with individual dysfunctional cognitions. Despite exposure being present in both groups, the intervention group showed a stronger symptom reduction, which may indicate an additional contribution of the confrontation with dysfunctional cognitions beyond general exposure, although differential exposure intensity cannot be ruled out. Regression analyses further indicated that increases in self-esteem and decreases in dysfunctional social phobia cognitions were associated with symptom reduction, but effect sizes were small (*R*^2^ = 0.054). These findings provide preliminary evidence for a possible cognitive mechanism; however, causal conclusions cannot be drawn, and other factors may also contribute to the observed changes. Future research should aim to disentangle the contributions of exposure and cognitive restructuring within avatar-based interventions.

To examine cognitive changes, we conducted exploratory analyses of SPK-scores as a manipulation check. The exploratory analyses showed a moderate reduction in the intervention group (Cohen’s *d* = 0.60, *t*(117) = 6.53, *p* < .001) and a smaller reduction in the control group (Cohen’s *d* = 0.21, *t*(116) = 2.23, *p* = .028). Although the control group showed significant changes, the effects were more pronounced in the intervention group. This suggests that the avatar-based intervention may be associated with a stronger reduction of dysfunctional thoughts, consistent with the assumed mechanism of cognitive change, although this conclusion remains exploratory. However, since the differences between the groups were small and both groups showed improvements, the results should be interpreted with caution. The observations in the control group suggest that other factors, such as general engagement, or psychoeducation may also contribute to the reduction of dysfunctional thoughts.

Taken together, these observations highlight the need for future research to examine the specific contributions of each of these elements (e.g. cognitive restructuring, psychoeducation or exposure), to better understand their individual and combined effects.

Additionally, the use of a subclinical sample, while providing an initial effectiveness assessment, may not reflect the full potential of the intervention. The exploratory analysis suggests that intervention is more effective for participants with higher severity of symptoms. Future research should focus on clinical populations to enhance external validity and test whether the intervention is more effective in individuals with significant social phobia symptoms.

A methodological issue is the short time frame used in the SPIN assessments, which measured symptoms within the same week, potentially skewing results. Extending the intervention period or increasing the number of sessions could address this. Deng et al.^48^ suggested a dose-response relationship between the number of sessions and effect size in treatments, which should be explored further in internet-based interventions for social anxiety.

Some technical challenges included participants not being able to return to the questionnaire after the exercise. While email communication helped prevent some issues, the lack of real-time support and control over environmental factors (e.g., noise, lighting) could have affected the intervention quality. However, the anonymity and flexibility of online participation, supported by studies on internet-based interventions^49^, suggest that such approaches can be resource-efficient and effective.

The dropout rate in this study was likely influenced by technical difficulties and the time commitment required for five measurement points. Some participants did not respond to follow-ups or lacked computer access, suggesting a smartphone-based application could improve participation. Due to data protection, exact reasons for dropout are unknown, but providing anonymous feedback options in future studies could help identify causes. Another potential reason is that confronting personal dysfunctional thoughts may have been demanding, as such interventions can trigger negative emotions^50^. Apart from this, no adverse effects or harms were observed. Still, future studies could include standardized assessments of adverse effects to evaluate potential risks and harms.

Avatar personalization could mitigate dropout by enhancing emotional engagement. In this study, all participants used the same female avatar, but offering customization options might improve identification with the avatar, leading to better outcomes^51,52^. Personalized avatars can foster a sense of autonomy, potentially increasing commitment to the intervention. The emotional engagement and effectiveness of virtual interventions are linked to participants’ sense of immersion and presence^53^. A decline in perceived presence might explain lower effect sizes. Future studies should explore enhanced avatar customization to boost engagement.

The Mood-State Hypothesis suggests that negative moods make dysfunctional beliefs more accessible for therapy^54^. Some participants may have found it difficult to articulate their automatic thoughts, indicating that priming them into a negative mood could enhance the intervention’s effectiveness.

Finally, the intervention allowed participants to face fears in a “safe space” by reducing safety behaviors^55^. As participants responded to the avatar, they may have learned that the anticipated negative outcomes did not occur, which could have contributed to reductions in symptoms and beliefs in dysfunctional cognitions.

## Conclusion

The present study suggests that an avatar-based intervention involving repeated exercises challenging dysfunctional beliefs is associated with reductions in social anxiety symptoms in individuals with subclinical SAD. Changes in self-esteem and social phobia-related cognitions were also associated with improvements in symptoms of SAD, although causal relationships cannot be concluded. Future research could examine the intervention in larger or clinical samples, include a no-intervention control group, address technical limitations, and investigate which specific features of the avatar contribute most to therapeutic outcomes.

## Supplementary Information

Below is the link to the electronic supplementary material.

Supplementary Material 1

## Data Availability

The datasets generated for this study are available on request to the corresponding author.
