# Supplementary material for: Effects of change in dysfunctional beliefs and self-esteem in avatar-based cognitive therapy for symptoms of social anxiety disorder: a randomized parallel trial
Source: Sci Rep. 2026 Feb 12;16:6144. doi: 10.1038/s41598-026-39641-x (PMC12901003; doi:10.1038/s41598-026-39641-x)
Supplement: Supplementary file 1 — Supplementary Material 1 [file 41598_2026_39641_MOESM1_ESM.docx]

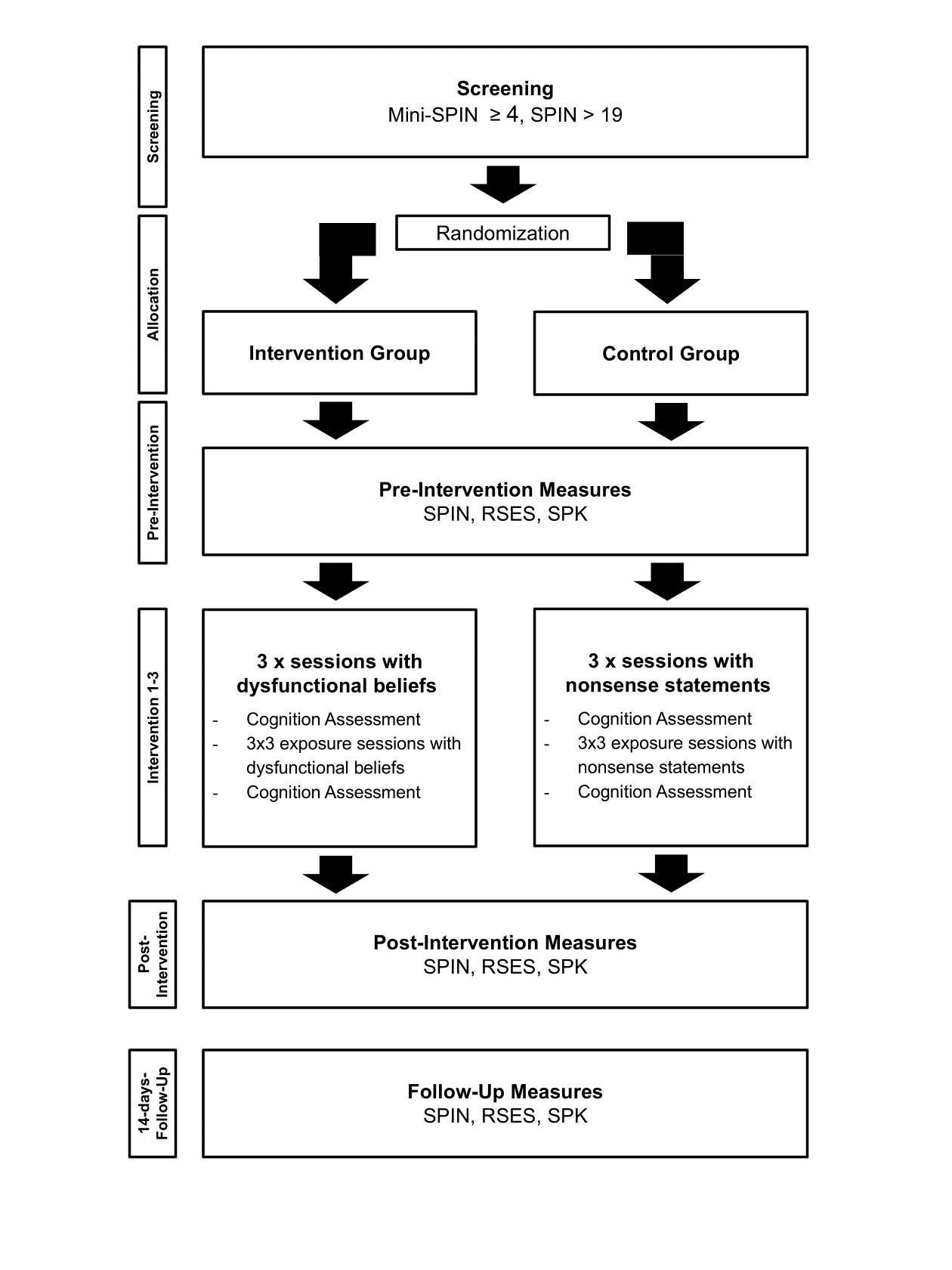


**Supplementary Figure S1.** Flow chart of the study's experimental procedure. *Note. Mini-SPIN = Mini - social phobia inventory, SPIN = Social Phobia Inventory, RSES = Rosenberg Self Esteem Scale., SPK = Social Cognitions Questionnaire*

**
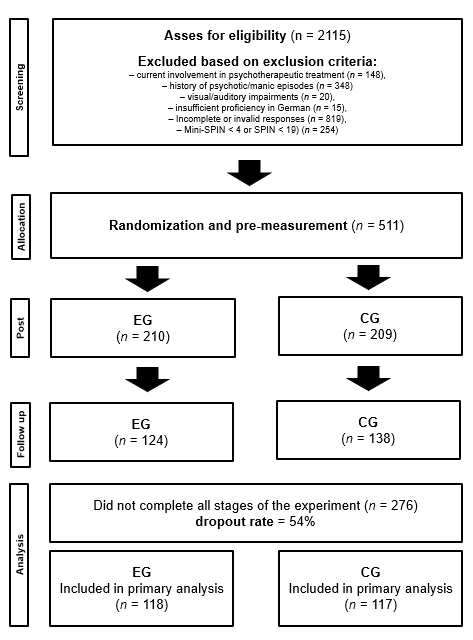
**

**Supplementary Figure S2.** Flow chart of participant progression through the study, including numbers of participants at each stage, exclusions and dropouts. *Note. EG = intervention group, CG = control group.*
